# *Helicobacter pylori*: Emergence of a Superbug

**DOI:** 10.3389/fmed.2014.00034

**Published:** 2014-10-14

**Authors:** Amin Talebi Bezmin Abadi

**Affiliations:** ^1^Department of Bacteriology, Faculty of Medical Sciences, Tarbiat Modares University, Tehran, Iran

**Keywords:** *Helicobacter pylori*, superbug, infection, treatment, virulence factors

## Introduction

It has been shown that *Helicobacter pylori* is highly genetically heterogeneous. A chromosome containing approximately 1,550 genes resulted in a mutant bacterial strain that adversely affects human health. Previously, regarded as a treatable infection, *H. pylori* is becoming harder to eradicate. The unique features in *H. pylori* biology such as high genetic variability, survival in the harsh conditions of the stomach, and special nitrogen metabolisms have triggered general research interest on this persistent infection. Interestingly, the notion “historic taboo” of sterility of the stomach has been changed after the successful *H. pylori* isolation by Marshall and Warren in 1983 (1). It is quite obvious that *H. pylori* is emerging as an extraordinary microorganism, which deserves careful consideration. Altogether, such particular biological features of *H. pylori* infection led to many queries in the minds of gastroenterologists, microbiologists, and even basic biologists, to spend thousands of hours applying for grants to study *H. pylori* (2). Current knowledge of the *H. pylori* virulence factors particularly after the latest findings in 2012 and 2013 called for new research on *H. pylori* and its clinical aspects (3–5). It is a general belief that certain *H. pylori* virutypes should be linked to specific gastroduodenal diseases, but recent studies draw an opposite conclusion. Actually, it is rather the treatment for *H. pylori* that is associated with bacterial virulence. The difficulty observed in *H. pylori* therapy is due to unpredictable antibiotic susceptibility results in various regions, unavailability of new effective drugs, and eventually lack of patient compliance during treatment. In brief, providing a new updated definition of *H. pylori* infection represents a shortcoming in gastrointestinal research. In this article, we propose realistic views on *H. pylori* therapy and virulence according to the latest findings with focus on a new definition of diseases caused by *H. pylori*.

## *H. pylori*: Old Born but Newly Recognized as a *Superbug*

It appears that *H. pylori* knew what to do to become a successful human gastric colonizer and we are just beginning to understand these mechanisms. Hence, it is worthwhile to consider new strategies to win the battle against this rogue microorganism. The *H. pylori* superbug contains unique biological features that enable it to survive safely in harsh environments. While some scientists insist on beneficiary impact of *H. pylori* colonization, others disagree. Nonetheless, current evidence suggests that the pathogenic *H. pylori* phenotype is more prevalent (6). Altogether, dealing with this bacterium (*superbug*) will be tough and time consuming. There is no doubt that improved therapeutic strategies can only be implemented if we acknowledge *H. pylori* as a superbug.

## Antibiotic Therapy of *H. pylori*: Defeated Strategy?

Undoubtedly, the emergence of resistant *H. pylori* strains only few years after its discovery by Warren and Marshall was an underestimated event (7–9). Antibiotic therapy was the first choice against *H. pylori* due to the direct association between remission of digestive diseases and successful bacterial eradication. In 1993, due to several observations of *H. pylori* resistant cases, a standard treatment was suggested to provide a well-established therapeutic regimen (10). Standard treatment for *H. pylori* infection involves clarithromycin, metronidazole, or amoxicillin, in conjunction with a proton pump inhibitor (PPI). This approach was originally a promising solution for reported *H. pylori* antibiotic resistance (11). Such a complex combination of drugs to cure the colonized infection has launched extensive efforts to identify an ideal simpler successful therapeutic intervention. Unfortunately, a steady increase in *H. pylori* resistance to clarithromycin, amoxicillin, and especially metronidazole resulted in the diminished use of standard therapy (12). Nevertheless, Zullo et al. suggested a new “sequential therapy” approach against reported treatment failures (13). They proposed using a PPI and amoxicillin for 5 days followed by a PPI with metronidazole and clarithromycin for the next 5 days (13). Despite various recommendations of suggested treatment regimens, an achievement of successful eradication was not possible, and some patients remained *H. pylori* positive lifelong. Until today, whenever a colonized patient is referred to a gastroenterologist, he/she will be prescribed the common *H. pylori* therapy, an old strategy that needs major reconsideration. In other words, globally increasing antibiotic resistance among *H. pylori* isolates is the best evidence for the necessity of new therapeutic strategies (14–18). Even if antibiotic resistance is not a potential problem, patient compliance can result in lower efficacy of *H. pylori* therapeutic regimens. We need to rethink current treatment strategies regarding this rogue bacterium. Actually, the frequency of resistant strains has now increased. Currently, *H. pylori* antibiotic resistance is much more problematic than 30 years ago. Conversely, the prevalence of *H. pylori* infection compared with 30 years ago is decreasing. More recently, it has been shown that the presence of various genetic elements such as plasmids and phages can explain *H. pylori*’s fascinating genetic diversity (19). Obviously, such characteristic of *H. pylori* infection call for an extraordinary adopted strategy on how to deal with this bacterium, which we may call a “*superbug.”* Looking back in the literature can show that we have never been confronted with such a complex therapeutic regimen for any bacterial or viral infection, except for *Mycobacterium tuberculosis* and HIV infection. Considering *H. pylori* as a superbug can be a starting point to investigate all possible mutations involved in antibiotic resistance in greater details. However, the point mutations in *H. pylori* genome are known to be responsible for antibiotic resistance. In the near future, new non-invasive and industrialized tests can help physicians detect mutations present in each isolated strain. Accordingly, prescription based on the determined mutational profile can increase the chance of successful treatment. Therefore, a better designed antibiotic therapy for *H. pylori* will soon become a reality in clinical practice. During the last decades, several new virulence factors have been characterized; however, we have still not identified one specific *H. pylori* virulence factor that covers the whole disease spectrum (20, 21). It is worthwhile mentioning that new rapid sequencing technology is providing accurate information about *H. pylori* genome and in depth gene structure (4, 22, 23), validating the present hypothesis about the association between *H. pylori* virulence factors and gastroduodenal diseases. As with *H. pylori* therapy, scientists are facing a new challenge in *H. pylori* virulence factors. At first glance, we are getting close to discovering the real virulence factor for *H. pylori*, but on the other hand, we should pay attention to the fact that the bacterium harbors a chromosome with more than 1550 genes of which most are not functionally characterized. Remarkably, our current understanding of *H. pylori* virulence factors poses a serious dilemma. There is not enough evidence to use virulence factors as clinically tools. Further work is required to find a relevant virutype to predict diseases outcome. Possibly, by the end of 2014, newer detailed information can ameliorate the current discussed dilemmas in *H. pylori* research. Applying a new definition of *H. pylori* infection as a superbug in human stomach, and placing higher priority on microbe–human interaction research, can provide more useful perspectives. Hopefully, these perspectives will enable researchers to answer frequently asked questions regarding *H. pylori* in the near future.

## Conflict of Interest Statement

The author declares that the research was conducted in the absence of any commercial or financial relationships that could be construed as a potential conflict of interest.
